# A novel deployable microstent for the treatment of glaucoma

**DOI:** 10.1016/j.xinn.2025.100935

**Published:** 2025-04-29

**Authors:** Yunlan Zhang, Weijia Zhang, Yunfang Yang, Callum Cuttle, Liam Morrow, Chun Zhang, Zhong You, Jared Ching

**Affiliations:** 1 Department of Engineering Science, University of Oxford, Oxford OX1 3PJ, UK; 2 Fariborz Maseeh Department of Civil, Architectural and Environmental Engineering, The University of Texas at Austin Cockrell School of Engineering, Austin, TX 78712, USA; 3 Department of Ophthalmology, Peking University Third Hospital, Beijing 100191, China; 4 Beijing Tsinghua Changgung Hospital Eye Center, School of Clinical Medicine, Tsinghua Medicine, Tsinghua University, Beijing 102218, China; 5 Beijing Visual Science and Translational Eye Research Institute (BERI), Beijing 102218, China; 6 John Van Geest Centre for Brain Repair, University of Cambridge, Cambridge CB2 0PY, UK; 7 Moorfields Eye Hospital, London EC1V 2PD, UK

Dear Editor,

Glaucoma is the second leading cause of blindness worldwide, after cataracts, and results in irreversible optic nerve damage and vision loss.^1^ The most common subtype, primary open-angle glaucoma (POAG), is caused by impaired aqueous humor (AH) drainage through the trabecular meshwork. POAG progresses gradually, often without symptoms until significant vision loss occurs. Treatments focus on lowering IOP using medicated eye drops, laser therapy, or surgical interventions. In the past decade, minimally invasive glaucoma surgery (MIGS) has emerged as a safer alternative to traditional surgeries, aiming to reduce IOP with minimal scleral or conjunctival disruption and a lower risk of fibrosis. MIGS devices improve AH drainage through the SCS, Schlemm’s canal, or the suprachoroidal space (Figure 1A). Among these approaches, tubular implants that drain from the anterior chamber (AC) to the SCS have demonstrated the most significant IOP reduction.^2^^,^^3^ Constructed from soft, biocompatible materials such as porcine gelatin and poly(styrene-block-isobutylene-block-styrene), these devices emulate traditional trabeculectomy surgery by forming a subconjunctival bleb, which is known for its potent IOP-lowering capabilities.^4^ However, clinical studies^5^ have revealed that the long-term effectiveness of these implants falls short of trabeculectomy due to issues like fibrosis and the eventual loss of bleb function, even with the use of anti-fibrosis medications.^6^ Additionally, these devices are susceptible to breakage and migration over time.

**Figure 1:**
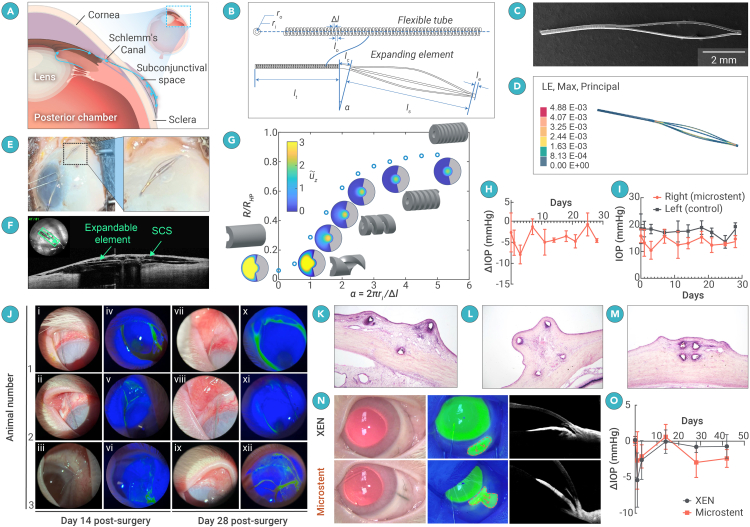
Design, fabrication, and experimental testing of the deployable microstent (A) A sagittal schematic of the eye’s anterior segment, demonstrating the anatomical placement of the microstent, whereby Aqueous humour is diverted from the AC to the SCS through the flexible tube, creating a subconjunctival bleb supported by the expanding element. (B) The key geometric parameters of the microstent. (C) Scanning electronic microscopy image of a deployable microstent sample. (D) Finite element (FE) simulation indicates that the expandable element remains 0.7 mm high under pressure inside the SCS. (E) A human cadaveric eye with the microstent being implanted into the SCS. The enlarged image demonstrates the formation of a subconjunctival bleb. (F) Anterior segment optical coherence tomography (AS-OCT) demonstrates the compliance of the expandible elements of the microstent and that the subconjunctival bleb is mechanically supported through the separation of the sclera and conjunctiva. (G) Normalized resistance of a helical tube as a function of coil spacing density α, computed numerically. Inset: profiles of normalized axial velocity ${\tilde{u}}_{z}$ (see color bar) are shown in a single transverse cross section for α = 0, 0.5, 1, 1.5, 2, 3.5, and 5 (left to right). Gray regions are the solid walls of the tube. (H) Mean delta IOP plotted over time. (I) Mean ± SD ΔIOP shows a trend of IOP reduction in microstent-implanted eyes vs. controls. (J) Anterior segment photography on post-operative days 14 and 28. (i–xii) The microstent implanted in the AC on day 14, showing a diffuse bleb and mild to moderate conjunctival injection in bright-field images with clear corneas, deep ACs, and clear crystalline lenses (i–iii). Note that, due to photophobia, animal 3 was not able to tolerate illumination for photography (iii). Topical 2% fluoresceine did not show any aqueous leak or keratopathy (iv–vi). On day 28 post surgery, clinical observations remained stable, including the presence of a diffuse subconjunctival bleb (vii–ix) and no changes with 2% fluorosceine (x–xii). (K–M) Histopathological laser-cut sections of the microstent implanted in the New Zealand White rabbit SCS, observed under a 10× objective lens, show transverse sections at different locations along the three microstents. In all cases, the struts appear well separated, indicating successful support of the SCS. Mild fibrosis is present along the struts. (N) The left panel shows color anterior segment photograph 28 days post surgery with the microstent and XEN visible in the superotemporal quadrant in the right eye with a subconjunctival bleb present. The middle panel shows subconjunctival flow of fluoresceine when the AC is injected. The right panel demonstrates anterior segment OCT of the same animals, demonstrating the position of the microstent and XEN, respectively. (O) Mean ± SD ΔIOP, demonstrating a direct comparison of IOP change between XEN and the microstent.

To address these limitations, we aimed to develop a novel, deployable microstent that can be delivered minimally invasively via a needle and subsequently expand within the SCS. This design incorporates structural elements to sustain conjunctival-episcleral separation without relying on anti-fibrosis treatments. It is specifically optimized to form a posterior, consistently elevated bleb while preventing migration, improving durability, and ensuring long-term efficacy.

Our device is constructed from nitinol, a biocompatible metal renowned for its proven long-term ocular safety and successful use in larger filtration devices like the EX-PRESS.^7^ Despite its common application in medical stents, nitinol has yet to be employed in microscale MIGS to create outflow to the SCS, primarily due to its stiffness relative to that of ocular tissues. By utilizing the superelasticity of nitinol and adopting a special structural shape, we successfully achieved deployability of our microstent. This innovation overcomes the challenges associated with current SCS-implanted MIGS devices while striving to match the effectiveness of trabeculectomy.

## Microstent

We leverage the concept of deployable structures and optimize the geometry at both micro- and macroscales to adjust the stiffness of the nitinol structure to match that of ocular tissues. This enhanced flexibility allows the structure to conform to surrounding tissues, potentially reducing fibrosis and minimizing patient discomfort. This microstent consists of a flexible tube that connects the AC to the SCS and a self-expanding element to support a subconjunctival bleb (Figures 1A and 1B). It is implanted via an *ab interno* approach using a clear corneal incision and deployed into the SCS to divert AH flow from the AC. A 25G needle (internal diameter, 260 μm) was used to load the microstent (outer diameter, 220 μm).

### Expandable element

The expandable element is designed to triple its original size upon deployment, a transformation enabled by the superelasticity of nitinol, which accommodates local strains up to 4% without permanent deformation. Laser cutting and shape-setting techniques were used to form the spindle-shaped expandable element (Figure 1C). It features four slender struts asymmetrically arranged along the central axis of the flexible tube. After deployment, these struts self-expand to lift the conjunctiva and Tenon’s layer from the sclera, creating a bleb. To minimize complications such as conjunctival erosion, blebitis, or infection, the design targets a 0.7-mm-tall space in the SCS, balancing effective drainage with reduced risk. The degree of expansion is influenced by factors like scleral compliance and conjunctival elasticity, as opposing forces from these tissues limit expansion.

Finite Element simulations guided the design, optimizing the microstent to achieve the desired expansion under a load of 243 Pa, derived from the rheological properties of hyaluronic acid-based ophthalmic viscosurgical devices.^8^ Results showed that a 1 mm expandable element can create a spindle-shaped bleb approximately 0.7 mm in radius and 6 mm in length without permanent deformation (Figure 1D). We also developed an additional FE model to analyze the strain field of the deployable microstent during the packing process. The analysis revealed a maximum strain of 0.015, which is 62.5% lower than the threshold for permanent deformation. Using cadaveric eyes, we refined the surgical approach and evaluated the microstent’s anatomical suitability. Prototypes with varying dimensions were successfully implanted via an *ab interno* approach, achieving proper positioning without impeding eyelid closure. Anterior segment optical coherence tomography (AS-OCT) imaging confirmed the correct subconjunctival placement, adherence to globe curvature, and bleb formation, validating its potential for MIGS in humans (Figures 1E and 1F).

### Flexible tube

Nitinol, though widely used for its biocompatibility, is significantly stiffer than human tissues. For example, a solid-walled nitinol tube has a bending stiffness of 4.24E−03 N⋅mm, nearly 3,000 times greater than scleral tissue (∼1E−06 Nmm). To address this, we reduced the stiffness by cutting a helical pattern into the tube, using a fixed cutting width of Δl = 0.03 mm and coil spacing of $l_{0}$ = 0.04 mm. This reduced the bending stiffness to 2.41E−06 Nmm, allowing the tube to conform to the scleral curvature. While this study focused on helical patterns, other porous or cellular designs could achieve similar results.

Increasing flexibility raised concerns about maintaining appropriate hydraulic resistance to balance AH outflow and prevent over- or underdrainage. AH flow is driven by IOP relative to episcleral venous pressure and opposed by resistance within the stent lumen, porous scleral tissue, and fibrosis over time. In the early postoperative phase, low resistance in the SCS can risk hypotony if the stent does not provide sufficient resistance. The Hagen-Poiseuille law (Equation 1) was used to estimate the maximum possible IOP reduction by calculating the pressure drop for laminar flow through a uniform tube. Results indicated that the current design maintains an IOP only 1 mm Hg above bleb pressure, which is inadequate to prevent hypotony:

(Equation 1) $$\Delta p=\left(\frac{8\mu L}{\pi {r_{i}}^{4}}\right)Q\text{.}$$

Given that the microstent’s helical design departs from a uniform cylindrical geometry, the Hagen-Poiseuille law may not capture its hydraulic resistance. To address this, we developed a numerical model accounting for the non-axisymmetric cross section.^5^ We impose a fixed pressure gradient G=Δp/L along the axis of the tube and compute the hydraulic resistance per unit length of the tube, given by

(Equation 2) $$R=\frac{G}{Q}\text{.}$$

Simulations showed that increasing coil density (α) concentrates flow into the central lumen, raising resistance to approximately 85% of the Hagen-Poiseuille prediction for tightly spaced coils (α ≥ 3.5). This result suggests that resistance is largely preserved while reducing stiffness by orders of magnitude through helical cuts (Figure 1G). These findings identify a design range where the device achieves a balance between flexibility and effective hydraulic resistance, ensuring its suitability for IOP regulation and the design we demonstrate here within this region.

### Performance in cadaveric porcine eyes

To evaluate the effect of the deployable microstent on IOP, we designed and 3D-printed the testing setup and performed perfusion experiments on porcine eyes to measure real-time IOP changes pre and post implantation.^9^ Using a validated *ex vivo* perfusion system, a syringe pump was connected via a three-way valve and tubing to a 30G needle inserted into the porcine eye. IOP was recorded for at least 30 min at steady state (variation within ±0.1 mm Hg) before and after implantation. Results from five experiments demonstrate an average IOP reduction of 4.53 ± 1.42 mm Hg (*p* < 0.0001; coefficient of variation = 0.31).

### Performance in New Zealand White rabbits

A pilot study using three New Zealand White rabbits was conducted to evaluate the safety, biocompatibility, and effectiveness of the microstent. The surgical implantation was smooth and atraumatic, with no complications such as bleeding, choroidal hemorrhage, or ocular trauma. Subconjunctival blebs consistently formed post surgery, and AS-OCT imaging confirmed proper placement and bleb formation. Anterior segment photography on postoperative days 14 and 28 showed microstent position and a diffuse bleb. Topical 2% fluorescein did not show any aqueous leak (Figure 1J). The animals showed no systemic adverse effects or postoperative concerns, with weight and behavior remaining normal throughout the study. Postoperative IOP measurements showed a significant reduction, with the greatest mean decrease of −7.88 mm Hg (−43.65%) observed on day 3. The final mean delta IOP on day 28 was −4.59 mm Hg (−25.36%), demonstrating controlled IOP reduction without hypotony, IOP spikes, or device migration. The blebs remained diffuse, with gradual thickening and vascular changes over time (Figures 1H and 1I).

Histological analysis revealed mild inflammatory responses and minimal fibrosis around the nitinol microstent (Figures 1K–1M). Microscopy confirmed stable tissue separation by the struts, suggesting the device’s long-term stability and biocompatibility. These results indicate that the microstent is safe and effective for regulating IOP *in vivo* over 28 days.

### Comparative assessment of commercially available MIGS

To evaluate the microstent’s performance against an established MIGS device, we conducted a head-to-head study with 12 New Zealand White rabbits split into microstent and XEN groups without any anti-fibrotic medication. Implantations were smooth for both devices, with no complications such as bleeding, hypotony, or ocular trauma. Subconjunctival blebs formed post surgery, and no leaks were detected (Figure 1N).

Post-surgical monitoring showed no significant welfare issues, though two animals died from anesthetic complications on day 14. Bleb parameters, vascularity, and AC depth changes were similar in both groups, with no significant differences between them.

IOP reduction patterns differed. On days 1 and 3, the XEN group showed significant IOP reductions of −5.39 mmHg (−28.54%) and −2.62 mmHg (−14.07%), respectively, while the microstent group had smaller, non-significant reductions. However, from day 14 onward, the microstent achieved sustained and significant IOP reductions of −2.94 mmHg (−18.23%) on day 28 and −2.39 mmHg (−17.10%) on day 42, unlike the XEN, which showed non-significant reductions during the same period (Figure 1O). Fluorescein injection confirmed subconjunctival flow in all animals on day 28. These results suggest that the microstent sustains IOP reduction without fibrosis augmentation, outperforming the XEN gel stent in a normotensive rabbit model over time.

## Discussion

We developed a novel deployable microstent for MIGS applications, leveraging deployable structure concepts and biocompatible nitinol to mechanically separate ocular tissues in the SCS. We hypothesize that this mechanism plays an important role in early failure in XEN, which relies solely upon aqueous flow to maintain the patency of the bleb. Persistent intermittent flattening of the bleb due to ocular excursions and eyelid closure, for example, leads to direct contact between the conjunctiva and episclera, creating a pro-fibrotic environment. In comparison, the deployable microstent prevents direct conjunctival-episcleral contact and maintains IOP reduction beyond 2 weeks, while existing devices, including XEN and PRESERFLO, fail to do so in the same animal model.^10^ Further, the microstent is associated with limited inflammation and fibrosis on histological analysis.

Our design includes helical cuts to reduce stiffness while preserving hydraulic resistance. Computational modeling showed less than a 15% drop in resistance compared to a solid tube, ensuring effective IOP regulation. *Ex vivo* and *in vivo* studies demonstrated significant IOP reduction (∼43.65% early and ∼25.36% sustained over 28 days) without hypotony, device breakage, or migration. Histological analysis revealed minimal inflammation and fibrosis.

Head-to-head comparison with XEN confirmed similar safety profiles but superior sustained IOP reduction with the microstent (−18.23% at 28 days versus −5.37% for XEN). This innovative microstent addresses limitations of current MIGS devices in early rabbit studies, demonstrating effective and stable IOP modulation for 6 weeks. Compared to other marketed MIGS devices at the same stage in animal trials, our device has shown superior performance in the SCS. These promising results provide confidence for future clinical studies, though further research is needed to assess long-term efficacy in humans.

## Declaration of interests

Z.Y., J.C., Y.Z., and Y.Y. are patentholders as filed by Oxford University Innovation Limited (worldwide patent pending, application number 2210218.0, filed July 13, 2022).

## References

[1] Quigley H.A., Broman A.T. (2006). The number of people with glaucoma worldwide in 2010 and 2020. Br. J. Ophthalmol..

[2] Lee R.M.H., Bouremel Y., Eames I. (2020). Translating Minimally Invasive Glaucoma Surgery Devices. Clin. Transl. Sci..

[3] Green W., Lind J.T., Sheybani A. (2018). Review of the Xen Gel Stent and InnFocus MicroShunt. Curr. Opin. Ophthalmol..

[4] Kim Y., Lim S.-H., Rho S. (2022). Bleb Analysis Using Anterior Segment Optical Coherence Tomography and Surgical Predictors of XEN Gel Stent. Transl. Vis. Sci. Technol..

[5] Governatori L., Oliverio L., Mermoud A. (2024). PreserFlo MicroShunt versus trabeculectomy: an updated meta-analysis and systematic review. Graefes Arch. Clin. Exp. Ophthalmol..

[6] Song D.-S., Qian J., Chen Z.-J. (2019). Ologen implant versus mitomycin-C for trabeculectomy. Medicine.

[7] Hendrick A.M., Kahook M.Y. (2008). Ex-PRESSTM Mini Glaucoma Shunt: surgical technique and review of clinical experience. Expet Rev. Med. Dev..

[8] Dick H.B., Krummenauer F., Augustin A.J. (2001). Healon5 viscoadaptive formulation: Comparison to Healon and Healon GV. J. Cataract Refract. Surg..

[9] Yang Y., Zhang Y., You Z. (2022). 3D-printed enucleated eye holder for research and surgical training. Acta Ophthalmol..

[10] van Mechelen R.J.S., Wolters J.E.J., Herfs M. (2022). Wound Healing Response After Bleb-Forming Glaucoma Surgery With a SIBS Microshunt in Rabbits. Transl. Vis. Sci. Technol..

